# Physiological and Proteomic Analysis Responsive Mechanisms for Salt Stress in Oat

**DOI:** 10.3389/fpls.2022.891674

**Published:** 2022-06-15

**Authors:** Xiaojing Chen, Zhongshan Xu, Baoping Zhao, Yanming Yang, Junzhen Mi, Zhou Zhao, Jinghui Liu

**Affiliations:** ^1^Cereal Industry Collaborative Innovation Center, Inner Mongolia Agricultural University, Hohhot, China; ^2^National Outstanding Talents in Agricultural Research and Their Innovative Teams, Hohhot, China

**Keywords:** oat, salt stress, proteomic, label-free, differentially expressed proteins

## Abstract

Oat is considered as a moderately salt-tolerant crop that can be used to improve saline and alkaline soils. Previous studies have focused on short-term salt stress exposure, and the molecular mechanisms of salt tolerance in oat have not yet been elucidated. In this study, the salt-tolerant oat cultivar Vao-9 and the salt-sensitive oat cultivar Bai5 were treated with 6 days of 0 and 150 mmol L^−1^ salt stress (nNaCl:nNa_2_SO_4_ = 1:1). Label-Free technology was then used to analyze the differentially expressed proteins in leaves under 0 and 150 mmol L^−1^ salt stress. The obtained results indicated that total of 2,631 proteins were identified by mass spectrometry in the four samples. The salt-tolerant cultivar Vao-9 mainly enhances its carbohydrate and energy metabolism through the pentose and glucuronate interconversions, and carbon fixation pathways in prokaryotes, thereby reducing the damage caused by salt stress. In addition, the down-regulation of ribosomes expression and the up-regulated expression of HSPs and CRT are all through the regulation of protein synthesis in response to salt stress. However, GABA metabolism presents a different synthesis pattern in Bai5 and Vao-9. The main KEGG function of differential expressed protein (DEP) in Bai5 is classified into protein processing in the endoplasmic reticulum, estrogen signaling pathway, antigen processing and presentation, longevity regulating pathway-multiple species, arginine and proline metabolism, beta-alanine metabolism, vitamin B6 metabolism, salmonella infection, chloroalkane and chloroalkene degradation, and limonene and pinene degradation. Moreover, the main KEGG functions of DEP in Vao-9 are classified as ribosome and carbon fixation pathways in prokaryotes, pentose and glucuronate interconversions, GABA ergic synapse, and taurine and hypotaurine metabolism. The results obtained in this study provide an important basis for further research on the underlying mechanisms of salt response and tolerance in oat and other plant species.

## Introduction

Soil salinization has become an important agricultural issue, with the global saline-alkali land area covering approximately 800 million hectares. It is estimated that, globally, soil salinity affects 80 million hectares of cultivated land (Zhang et al., 2012). Previous studies have reported that salinity is one of the main factors restricting the growth of crops (Flowers and Colmer, 2008), because it can cause ion imbalance, hyperosmotic stress, and oxidative damage in plants (Mahajan and Tuteja, 2005; Chinnusamy et al., 2006; Tuteja, 2007). To prevent the potentially harmful effects of such stresses, plants have evolved sophisticated mechanisms to recognize external signal networks and serve as evidence for adaptive responses at the physiological, biochemical, and molecular levels (Nam et al., 2017).

In recent years, sequencing of many plant genomes has been completed, which has led to many researchers exploring the function of various genes. Several previous studies have identified and cloned some proteins such as osmotic pressure-synthesizing protein (Verbruggen et al., 1993), ion channels (Horie et al., 2001), signal transduction pathways, and other important genes of enzymes associated with salt stress (Zhu, 2002; Hu et al., 2006). The studies have revealed the basic functions of these genes in response to salt stress. However, the mRNA levels are usually not associated with the protein expression levels due to the variable splicing of transcription and post-translational modifications (such as phosphorylation and glycosylation). It is worth noting that the protein expression levels are more directly associated with signal transduction and metabolic processes under salt stress. Therefore, it is important to study salt stress response at the protein level.

Analyzing the salt-responsive proteome in plants provides more information for understanding the complex mechanisms of plant salt tolerance. Currently, about 2,100 salt-responsive proteins have been identified in the buds, leaves, roots, seedlings, radicles, and hypocotyls of arabidopsis (Pang et al., 2010), barley (Witzel et al., 2009; Rasoulnia et al., 2011), wheat (Peng et al., 2009; Jacoby et al., 2010), sugar beet (Wang et al., 2019) and other crops. The proteins are involved in changes targeting photosynthesis, active oxygen scavenging system, ion homeostasis, osmotic homeostasis, membrane transport, signal transduction, protein synthesis, and other pathways. This general information has laid a solid foundation for further research on the molecular mechanism of salt tolerance in plants. However, a previous study reported that different crops or varieties of the same crop have different salt tolerance mechanisms (Guo et al., 2012).

Oat (*Arena sativa* L.), an annual gramineous herb used as a food and feed crop, has the characteristics of salt-alkali tolerance, barren tolerance, and cold resistance. It has become a pioneer crop for improving saline-alkali land. Presently, the research on oats salt tolerance mainly focuses on ion absorption and accumulation, physiological changes of oats mediated by exogenous substances (Gao et al., 2019), transcriptome (Wu et al., 2018), metabolome (Xu et al., 2021) and proteome. However, only a single species has been selected for research (Bai et al., 2016), and thus the molecular mechanism of salt tolerance between the species has not yet been elucidated. The cultivation of oats in China is mainly concentrated in the semi-arid farming and pastoral areas in the northwest and the high-altitude mountainous areas in the southwest, with Inner Mongolia province having the largest planting area. The saline soil of Inner Mongolia mainly contains Na^+^, K^+^, Cl^−^, and SO_4_^2−^ salt ions, and the ratio of Cl^−^ to SO_4_^2−^ is about 1. Therefore, the Label-Free technology was used in this study to compare the leaves of salt-sensitive varieties (Bai5) and salt-tolerant varieties (Vao-9) under 150 mmol L^−1^ (nNaCl: nNa_2_SO_4_ = 1:1) salt stress. In this study, we address two questions: (1) What are the proteins that are involved in the oat salt stress response? (2) What biological processes/pathways are these proteins involved in? The results obtained in this study will provide a more effective scientific basis and theoretical basis for crop salt tolerance breeding.

## Materials and Methods

### Plant Culture and Salt Treatment

The salt-tolerant oat cultivar Vao-9 selected by Ottawa Research and Development Centre, Agriculture and Agri-Food Canada and the salt-sensitive variety Bai5 were obtained from Baicheng Academy of Agricultural Sciences (Xu et al., 2021). This study was conducted in the greenhouse at the Oat Research Center of Inner Mongolia Agricultural University. Thirty seeds of each variety were sown in a plastic bucket (the upper diameter of the bucket was 24 cm, the lower diameter was 22 cm, and the height was 25 cm) filled with the substrate (sand: vermiculite: ceramsite = 3:1:2). After emergence, the seedlings were thinned to 20 plants per bucket. It is worth noting that five round holes with a diameter of 4 mm were drilled at the bottom of each bucket for water flow and ventilation. The plants were fed with 250 ml of Hogland nutrient solution three times a week. The oats were then treated with 100 and 150 mmol L^−1^ salt stress at the three-leaf stage (molar concentration NaCl:Na_2_SO_4_ = 1:1 mixed in the nutrient solution) for 6 days, and the same volume of nutrient solution was used for the control plants. This was followed by the sample collection process where 2 g leaf samples for each treatment and the control were transferred to 1.5 ml cryopreservation tubes. Incubation conditions were 16 h of light at 25°C, 8 h of darkness at 20°C, humidity at 70%. The samples were then quickly frozen in liquid nitrogen, and stored at −80°C for physiological index detection. Proteomics analysis was then conducted for CK and 150 mmol L^−1^ processed samples.

### Physiological Analysis

For ion concentration determination, root samples were dried in an oven at 70°C for 3 days, then digested in a concentrated nitric acid at 140°C. K^+^, Na^+^, Ca^2+^, and Mg^2+^ contents in the digested solution were determined using an inductively coupled plasma-optical emission spectrometer (iCAP 6000 series, Thermo Fisher scientific, United States) as per the manufacturer’s instructions. Superoxide Dismutase(SOD), Peroxide(POD), malondialdehyde (MDA) and proline content were measured using Assay Kit A001-1-1, A084-3, A005, A123, A003-1, and A145, respectively, (Nanjing Jiancheng Bioengineering Institute, China). We performed One-Way ANOVA analysis using the SPSS software (IBM SPSS Statistics Version19.0) to associations of different index between the treatments. Value of *p* ≤ 0.05 and *p* ≤ 0.01 were considered significant and highly significant, respectively.

### Extraction of Total Protein From Oat Leaves

The frozen samples were crushed using a crusher pre-cooled with liquid nitrogen, and liquid nitrogen was then used to grind the crushed powder. The powder was added to the lysis buffer [100 mM NH4HCO3(pH 8),6 M Urea and 0.2% SDS] according to 1:10 (w/v) ratio, followed by vortexing. Ultrasound was then conducted for 60 s at 0.2 s on, followed by 2 s off at 22% amplitude. The proteins were then extracted at room temperature for 30 min, followed by centrifugation at 15,000 × *g* for 1 h at 10°C. The supernatant was then collected, divided, and frozen at −80°C after loading.

### Protein Quantification

The Bradford (1976) method was used to determine the protein concentration of each sample. The protein concentration of each sample (μg μl^−1^) was calculated according to the curve formula. BSA standard protein solutions and sample solutions with different dilution multiples were added into 96-well plate to fill up the volume to 20 μl, respectively. Each gradient was repeated three times. The plate was added 180 μl G250 dye solution quickly and placed at room temperature for 5 min, the absorbance at 595 nm was detected. The standard curve was drawn with the absorbance of standard protein solution and the protein concentration of the sample was calculated. Twenty microgram of the protein sample was loaded to 12% SDS-PAGE gel electrophoresis, wherein the concentrated gel was performed at 80 V for 20 min, and the separation gel was performed at 120 V for 90 min. The gel was stained by coomassie brilliant blue R-250 and decolored until the bands were visualized clearly.

### Proteolysis (Filter Aided Sample Preparation)

After protein quantification, 200 μg protein solution was transferred to a centrifuge tube followed by the addition of DTT to make a final concentration of 25 mmol L^−1^. The solution was then reacted at 60°C for 1 h, followed by the addition of iodoacetamide to make a final concentration of 50 mmol L^−1^. The solution was then kept at room temperature for 10 min. After reductive alkylation, the protein solution was added to a 10 K ultrafiltration tube, and centrifuged at 12,000 × *g* for 20 min. After centrifugation, the solution at the bottom of the tube was collected followed by the addition of 100 μl Dissolution buffer which containing 0.1 M triethylammonium bicarbonate (TEAB, pH 8.5) and 6 M urea. The solution was centrifuged at 12,000 × *g* for 20 min, and then the solution at the bottom of the collection tube was discarded and the process was repeated three times. Trypsin was then added to the new ultrafiltration tube to make a solution with a total protein mass of 4 μg (mass ratio to protein was 1:50) and volume of 50 μl. The reaction was then incubated overnight at 37°C. The next day, the solution was centrifuged at 12,000 × *g* for 20 min, and the peptide solution at the bottom of the tube after enzymatic digestion by centrifugation was collected. Fifty microliter dissolution buffer was then added to the ultrafiltration tube, followed by centrifugation at 12,000 × *g* for 20 min. The obtained solution at the bottom of the ultrafiltration tube was then combined with the solution in the previous step to obtain a total solution of 100 μl in the collection tube sample after enzymolysis. Finally, the solution was lyophilized in readiness for loading.

### Nano-Upgraded Reversed-Phase Chromatography-Q Exactive for Protein Analysis

Twenty microliter preconstituted 2% methanol and 0.1% formic acid were used for this experiment. The solution was centrifuged at 12,000 × *g* for 10 min, the supernatant drawn, and finally the sample was loaded. Ten microliter sample volume was used to load. The loading pump flow rate was 350 nl min^−1^ for 15 min, while the separation flow rate was 300 nl min^−1^.

### Mass Spectrometry Data Analysis

The database uniprot-Pooideae361804_20170619.fasta. Fasta (362,934 sequences) was used. Mass spectrometry analysis was done using a Thermo Q Exactive mass spectrometer. Peptide Spectrum Matches (PSMs) with more than 95% reliability were trusted PSMs, while the proteins which contains at least one unique peptide (specific peptides) were the trusted proteins. It is worth noting that this study only used trusted peptides and proteins, and FDR verification was used to remove peptides with FDR greater than 1% and egg whites. The protein was different between the pairs of samples to be compared in the different replicate groups, and the mean value of the different multiples was used as the multiple of the difference between the two samples. *T*-test was then used to obtain the value of *p*, which was used as the significance index.

### Data Processing and Bioinformatics Analysis

Microsoft Excel 2010 and SAS 9.0 software were used for the statistical analysis of all the data obtained in this study. Common functional database annotations were performed for the identified proteins, including COG, GO, and KEGG databases. A series of differential protein functional analysis such as GO and KEGG functional enrichment analysis were then performed for the selected differentially expressed proteins.

### Real-Time Quantitative Reverse Transcription PCR Analysis

All qRT-PCR experiments were run in triplicates on a Light-Cycler Roche 480 instrument (Roche Applied Science, Mannheim, Germany). Primers of choriolysin genes and actin were used as housekeeping genes (the primers shown in Table 1). mRNA level of choriolysin was estimated using 2^−ΔΔCt^ method. Data is presented as mean ± SEM. One-way ANOVA was used for data analysis.

**Table 1:** Quantitative qRT-PCR primers.

Name	Description	Gene	Sequence (5′–3′)	Length (bp)
A0A2S3GZF9	Dihydrolipoamide acetyltransferase component of pyruvate dehydrogenase complex	PAHAL_2G260000	F: TGGATGAAACTCTGCCAGCA	220
			R: GTCGAGCAAGGTCGTGAGTA	
M8A623	Aquaporin PIP1-1	TRIUR3_04548	F: AGCAGGCTGTTTGTTGGAGT	123
			R: GCAGAAGATGAGGAGAGGCC	
A0A2T7DAI6	PEROXIDASE_4 domain-containing protein	GQ55_6G283200	F: TTGTCGTTCTCCTTGAGGGC	275
			R: ATCGAGGACCTCAACTCCCA	
I1I9A3	PEROXIDASE_4 domain-containing protein	100839539	F: GCGCGCTTGCATGGTTATTA	160
			R: CAGGAGGAATACACCGGAGC	
A0A1D6QPT3	Phosphoenolpyruvate carboxylase isoform 1	ZEAMMB73_Zm00001d053453	F: ACAGGAATGAAGGAGCCAGAG	110
			R: ACACCATTACATACTTCCTGACACT	
A0A1J7HFP8	Phosphoglycerate kinase	TanjilG_12206	F: ATATTGCGGTGGGATCGACC	234
			R: TTTTCGCTGGTGTAAGCCCT	
K3XV32	Uncharacterized protein	SETIT_4G175200v2	F: CGTAGGGCAACTGGTGGATT	251
			R: TCAAGAAGCTCCAGGCCAAG	
M1AX28	Uncharacterized protein	102605963	F: TAGAAATGGAAGTCGCGGGC	177
			R: ACTTTCCCCACCCAAACTCG	
Actin	*Avena sativa* actin (ACT) mRNA, partial cds	MH260250.1	F: CCAATCGTGAGAAGATGACCC	135
			R: CACCATCACCAGAATCCAACA	

## Results

### Physiological Changes of Oat Leaves in Response to Salt Stress

**Figure 1:**
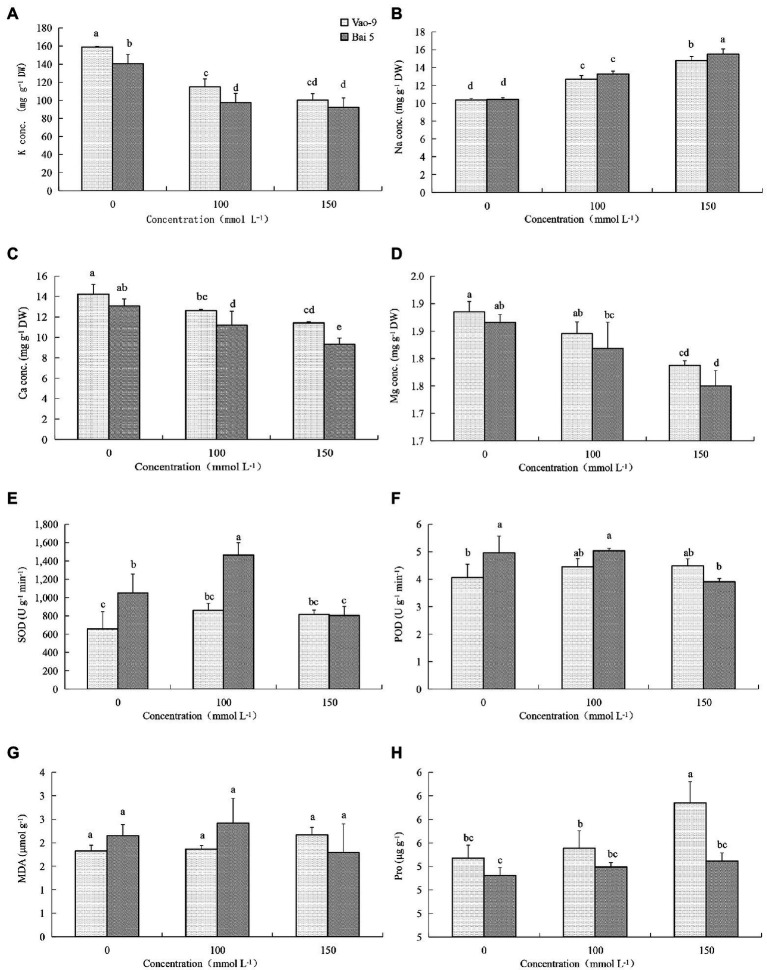
Physiological changes in the leaves of Vao-9 and Bai5 under normal and salt stress conditions. Contents of K^+^ **(A)**, Na^+^ **(B)**, Ca^2+^ **(C)**, Mg^2+^ **(D)**, SOD **(E)**, POD **(F)**, MDA **(G)**, and Pro **(H)** were determined in the leaves of Vao-9 and Bai5 after salt treatments and CK. Data are means ± SD of three biological replicates (*n* = 3) and different letters indicate significant difference at *p* < 0.05 by the One-Way ANOVA test.

The K^+^ content in leaves of two oat cultivars decreased with the increase of salt stress concentration (Figure 1). But the three concentrations showed that K^+^ content of Vao-9 was higher than that of Bai5, which were 12.9%, 18.2%, and 8.8%, respectively. With the increase of salt stress concentration, the Na^+^ content of the leaves of the two cultivars of oat showed a gradually increasing trend. The Na^+^ content of Bai5 was higher than that of Vao-9 under each concentration treatment, and the difference between the two cultivars was more significant under severe stress. The Ca^2+^ content in leaves of the two cultivars of oat decreased with the increase of salt stress concentration. The Ca^2+^ content of Vao-9 was higher than that of Bai5 under each concentration treatment, which were 8.8%, 12.8%, and 22.5%, respectively. The change trend of Mg^2+^ content in leaves of two varieties of oat was the same as that of Ca^2+^, and both decreased with the increase of salt stress concentration.

SOD enzyme activity of Vao-9 leaves increased with the increase of salt stress concentration, and the moderate and severe stress increased by 30.8% and 24.5% compared with CK, respectively; SOD enzyme activity of Bai5 leaves increased first and then decreasing trend. POD enzyme activity of Vao-9 increased but it increased first and then decreased in Bai5 with the increase of salt stress concentration. The variation trend of MDA content in oat leaves was consistent with SOD and POD. The content of MDA in Vao-9 leaves under moderate and severe stress increased by 2.7% and 19.2%, respectively, compared with CK. The content of Pro in leaves of two oat cultivars increased with the increase of salt stress concentration. The Pro content of Vao-9 was higher than that of Bai5 among all treatments, and only under severe stress showed differences among cultivars, which were 1.5%, 1.5%, and 4.6%, respectively.

### The Effect of Salt Stress on Quantitative Proteome

A total of 2,631 proteins including 2,471 in Bai5 and 2,493 in Vao-9 were qualitatively obtained by mass spectrometry using the Label-Free method. Among the obtained total proteins, 138 were specific in Bai5 while 160 were specific in Vao-9. In addition, 2,333 proteins were shared by the two varieties, accounting for 88.7% of the total number. The 2,631 proteins identified by mass spectrometry were screened according to the fold change >2 or <0.65 (*p* < 0.05), and a total of 262 differential expressed proteins (DEPs) were obtained. Among them, there were 76 proteins in Bai5 where 51 were up-regulated and 25 were down-regulated. On the other hand, 141 of the 214 proteins in Vao-9 were up-regulated while 73 were down-regulated. However, only 28 of the selected 262 DEPs were co-expressed in the two varieties, accounting for 10.7% of the total, which was far smaller than the 88.7% of the co-expressed proteins in the total protein. Specifically, 48 of the 262 DEPs were expressed in Bai5, while 186 were expressed in Vao-9. This accounts for 18.3% and 71%, respectively, of the total number of DEPs [the vast majority (89.3%) of DEPs]. Moreover, both groups of DEPs were specifically expressed in the two varieties. For the 28 DEPs co-expressed in the two varieties, 18 were up-regulated and 10 were down-regulated (Table 2).

**Table 2:** Number of proteins and DEPs identified from the samples (>2 or <0.65-fold change, *p* < 0.05).

	**Total**	**Bai5**	**Vao-9**	**Unique in Bai5**	**Unique in Vao-9**	**Overlap of Bai5 and Vao-9 (ratio to total)**
Protein	2,631	2,471	2,493	138	160	2,333 (88.7%)
	**Bai5**	**Vao-9**	**Total**	**Overlap of BY and V (ratio to total)**		
**Bai5**				**Vao-9**	**Total**
Protein up-regulated	51	141	174	18	18	18
Protein down-regulated	25	73	88	10	10	10
Cultivar unique	48	186	234	--	--	--
Total	76	214	262	28	28	28

### Hierarchical Cluster Analysis of DEPs

**Figure 2:**
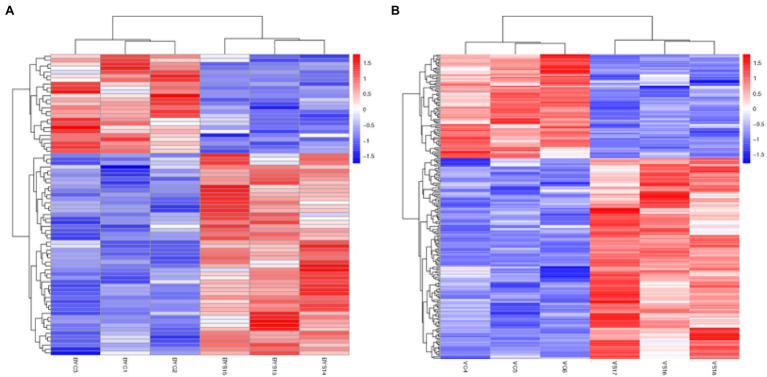
Hierarchical cluster analysis of the differential expressed proteins (DEPs). **(A)** Hierarchical cluster analysis of the DEPs in Bai5; **(B)** hierarchical cluster analysis of the DEPs in Vao-9. BYC1, BYC2, BYC3, represent CK samples with three replicates; BYS13, BYS14, BYS15, represent the treated (nNaCl:nNa_2_SO_4_ = 1:1) 150 mmol L^−1^ with three replicates; VC4, VC5, VC6 represent CK samples with three replicates; and VS16, VS17, VS18, represent the treated (nNaCl:nNa_2_SO_4_ = 1:1) 150 mmol L^−1^ with three replicates.

All the selected DEPs were analyzed using hierarchical clustering according to the obtained Label-Free protein abundance data. The 12 samples analyzed included the control and salt treatment for the two varieties, and the experiment was replicated three times. The obtained results indicated that the three samples each for BYC, BYS, VC, and VS were directly replicated, and there were significant differences between the treatment and CK. In addition, the changes in protein abundance obtained by cluster analysis not only illustrated the huge and complex changes at the proteome level, but also the diversity of expression levels of the two varieties after salt stress (Figure 2).

### Identification and Classification of DEPs

**Figure 3:**
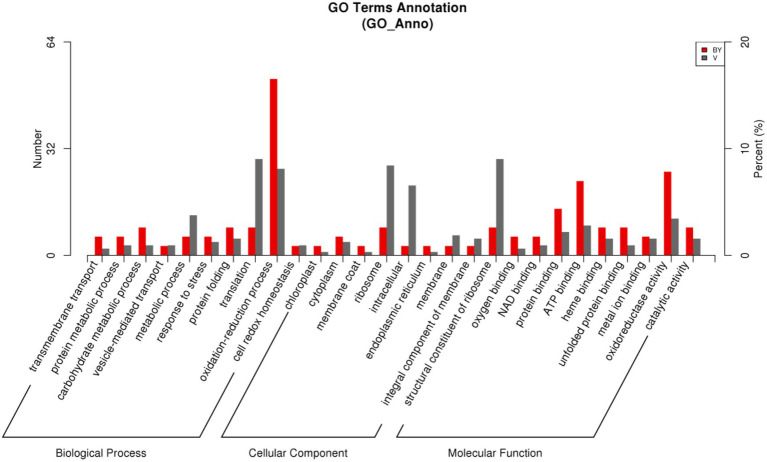
GO function classification of the DEPs.

According to the GO database, we used biological process (BP), cellular component (CC), and molecular function (MF) to perform functional analysis of the DEPs (Figure 3). The obtained results indicated that the functional annotations between the two varieties are similar. The main categories of BP were oxidation–reduction process and translation, while the main categories of CC were ribosome and intracellular. Furthermore, the main categories of MF were oxidoreductase activity, ATP binding, and structural constituent of ribosome. The main KEGG functions of the DEPs in Bai5 were classified into protein processing in the endoplasmic reticulum, estrogen signaling pathway, antigen processing and presentation, longevity regulating pathway-multiple species, arginine and proline metabolism, beta-alanine metabolism, vitamin B6 metabolism, salmonella infection, chloroalkane and chloroalkene degradation, and limonene and pinene degradation. On the other hand, the main KEGG function classification of the DEPs in Vao-9 were ribosome, carbon fixation pathways in prokaryotes, pentose and glucuronate interconversions, GABA ergic synapse, and taurine and hypotaurine metabolism.

**Figure 4:**
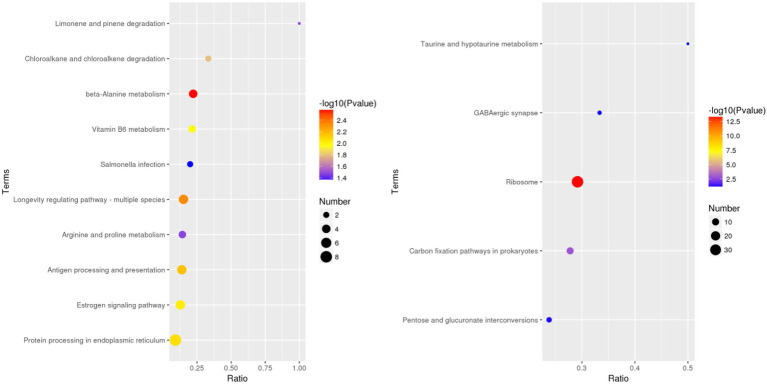
KEGG pathway enrichement analysis of the DEPs in Bai5 and Vao-9.

**Figure 5:**
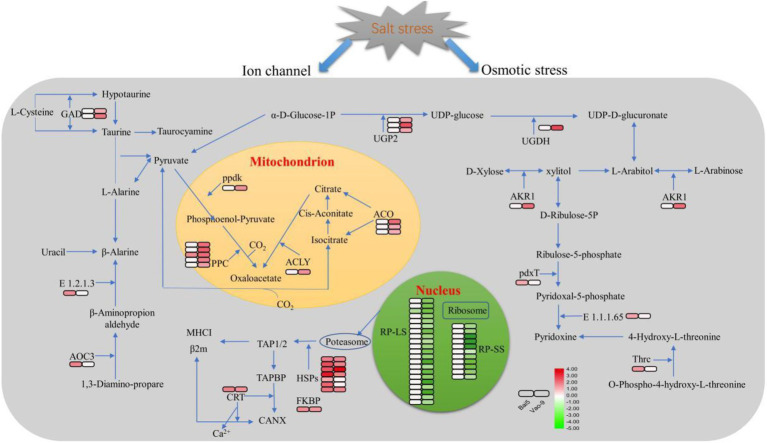
Schematic presentation of the critical salt stress responsive proteins in oats. The fold change of DEPs are indicated by color filled in the squars on the right (see color key). The left square represents the fold change in Bai5 and the right square represents the fold change in Vao-9. The particular definition and fold change of proteins are in Table 4.

In addition, the number of DEPs in the main functional categories in the two varieties were compared (Table 3). They include carbohydrate and energy metabolism, photosynthesis and electron transport chain, signal sensing and transduction, protein synthesis, and second metabolism. More proteins involved in carbohydrate and energy metabolism, protein synthesis and second metabolism were found in Vao-9 than in Bai5. Finally, the table shows candidate DEPs showing important functions or tissue-specific expression profiles in the two varieties, while the figure shows their relationship in the main functional categories (Figures 4, 5; Table 4).

**Table 3:** The numbers of DEPs from main functional categories in Bai5 and Vao-9.

Main categories	Subclass	Bai5 (up/down)	Vao-9 (up/down)
Carbohydrate and energy metabolism	1. Pentose and glucuronate interconversions	0 (0/0)	5 (5/0)
	2. Carbon fixation pathways in prokaryotes	0 (0/0)	10 (10/0)
Protein synthesis	1. Protein processing in endoplasmic reticulum	8 (8/0)	6 (6/0)
	2. Ribosome	0 (0/0)	35 (0/35)
	3. Antigen processing and presentation	5 (5/0)	3 (3/0)
	4. Estrogen signaling pathway	5 (5/0)	3 (3/0)
Stress defense and other stress-responsive proteins	1. GABAergic synapse	0 (0/0)	3 (3/0)
	2. Arginine and proline metabolism	3 (1/2)	0 (0/0)
	3. beta-Alanine metabolism	4 (2/2)	0 (0/0)
	4. Vitamin B6 metabolism	3 (3/0)	0 (0/0)

**Table 4:** The candidate DEPs from main functional categories in Bai5 and Vao-9.

No.	Description	Name	Fold change	
			BYS/BYC	VS/VC
**Carbohydrate and energy metabolism**				
I1HRS1	Phosphoenolpyruvate carboxylase	ppc	ns	4.001
D2T2H9	Phosphoenolpyruvate carboxylase	ppc	ns	4.853
F2CWA2	Phosphoenolpyruvate carboxylase	ppc	2.980	4.120
W5FCI5	Phosphoenolpyruvate carboxylase	ppc	ns	3.395
M0XEC5	Phosphoenolpyruvate carboxylase	ppc	ns	2.337
A0A1D6D8M0	Phosphoenolpyruvate-protein kinase (PTS system EI component in bacteria)	ppdK	ns	2.033
I1I0Y4	Aconitase A	ACO, acnA	ns	4.221
M0VQ49	Aconitase A	ACO, acnA	ns	2.041
M8CZ57	Aconitase A	ACO, acnA	ns	2.046
W5FDW8	UDP-glucose 6-dehydrogenase	UGDH, ugd	ns	6.264
I1ISL8	UDP-N-acetylglucosamine pyrophosphorylase	UGP2, galU, galF	ns	2.337
Q43772	UDP-N-acetylglucosamine pyrophosphorylase	UGP2, galU, galF	ns	5.493
W5FGH0	UDP-N-acetylglucosamine pyrophosphorylase	UGP2, galU, galF	ns	2.186
W5GEJ3	Aldo/keto reductase, related to diketogulonate reductase	E1.1.1.21, AKR1	ns	4.064
A0A1D5VL14	ATP citrate (pro-S)-lyase	ACLY	ns	2.826
**Proteinsynthesis**				
Q3I0N4	Molecular chaperone IbpA, HSP20 family	HSP20	3.529	3.186
I1GZ93	Molecular chaperone IbpA, HSP20 family	HSP20	5.804	3.147
I1IF07	Molecular chaperone IbpA, HSP20 family	HSP20	5.411	13.249
F4Y589	Molecular chaperone, HSP90 family	htpG, HSP90A	10.437	3.348
A0A1C6ZYA4	Molecular chaperone, HSP90 family	htpG, HSP90A	2.146	ns
F2DYT5	Molecular chaperone DnaK (HSP70)	HSPA1_8	4.442	ns
M8BCN0	Molecular chaperone DnaK (HSP70)	HSPA1_8	3.828	3.201
F2E3N4	FK506-binding protein 4/5	FKBP4_5	2.987	2.679
I1HU73	Calreticulin	CRT	3.318	2.855
A0A1D5RQS4	Ribosomal protein L1	RP-L1, MRPL1, rplA	ns	0.179
W5ECL2	Ribosomal protein L2	RP-L8e, RPL8	ns	0.161
I1GM81	Ribosomal protein L4	RP-L4e, RPL4	0.307	0.140
M0YWX9	Ribosomal protein L4	RP-L4, MRPL4, rplD	ns	0.238
F2E1T0	Ribosomal protein L5	RP-L5, MRPL5, rplE	ns	0.262
A0A1D5RV09	Ribosomal protein L7/L12	RP-L7, MRPL12, rplL	ns	0.341
I1HQ35	Ribosomal protein L7/L12	RP-L7, MRPL12, rplL	ns	0.141
M0WJN7	Ribosomal protein L7/L12	RP-L7, MRPL12, rplL	ns	0.254
I1IE72	Ribosomal protein L9	RP-L9, MRPL9, rplI	ns	0.335
A0A1D6DMG9	Ribosomal protein L10	RP-L10, MRPL10, rplJ	ns	0.243
I1HRK1	Ribosomal protein L13	RP-L13, MRPL13, rplM	ns	0.159
M7ZR36	Ribosomal protein L14	RP-L16, MRPL16, rplP	ns	0.229
I1H7Z7	Ribosomal protein L15	RP-L15, MRPL15, rplO	ns	0.085
I1GPZ1	Ribosomal protein L18	RP-L5e, RPL5	ns	0.324
M7ZME4	Ribosomal protein L18	RP-L18, MRPL18, rplR	ns	0.125
F2CSC5	Ribosomal protein L19E	RP-L19e, RPL19	ns	0.206
I1HZC3	Ribosomal protein L21	RP-L21, MRPL21, rplU	ns	0.074
R7W431	Ribosomal protein L21E	RP-L21e, RPL21	ns	0.306
I1IF27	Ribosomal protein L29	RP-L29, rpmC	ns	0.079
A0A1D5YHB0	Ribosomal protein L30/L7E	RP-L7e, RPL7	ns	0.298
M0WUC6	Ribosomal protein L31	RP-L31, rpmE	ns	0.302
W5E6V3	Ribosomal protein S1	RP-S1, rpsA	ns	0.282
D7F3Z0	Ribosomal protein S3	RP-S3, rpsC	ns	0.255
M8D3H8	Ribosomal protein S4E	RP-S4e, RPS4	ns	0.058
A0A1D8KWK8	Ribosomal protein S4 or related protein	RP-S4, rpsD	ns	0.060
A0A1D5XLA5	Ribosomal protein S5	RP-S5, MRPS5, rpsE	ns	0.064
M8BR59	Ribosomal protein S5	RP-S2e, RPS2	ns	0.455
M7YI57	Ribosomal protein S6	RP-S6, MRPS6, rpsF	ns	0.226
M8B4K5	Ribosomal protein S8	RP-S15Ae, RPS15A	ns	0.133
A0A1D5TUK4	Ribosomal protein S8E	RP-S8e, RPS8	ns	0.144
I1GMV8	Ribosomal protein S9	RP-S9, MRPS9, rpsI	ns	0.211
A0A1D5U621	Ribosomal protein S13	RP-S18e, RPS18	ns	0.351
**Stress defense and other stress-responsive proteins**				
C5IW59	Glutamine synthetase	glnA, GS	ns	2.339
I1I7Q1	Glutamate decarboxylase	E4.1.1.15, gadB, gadA, GAD	ns	2.008
A0A1D5YAL5	Glutamate decarboxylase	E4.1.1.15, gadB, gadA, GAD	ns	3.845
A0A1D5UEP5	Cu^2+^-Containing amine oxidase	AOC3, AOC2, tynA	3.226	ns
I1HWV1	Glutamine amidotransferase PdxT (pyridoxal biosynthesis)	pdxT, pdx2	2.135	ns
T1MRH6	Predicted oxidoreductase (related to aryl-alcohol dehydrogenase)	E1.1.1.65	2.265	ns
A0A1D5XXT6	———	MPAO, PAO1	0.353	ns
A0A1D6CAG8	———	MPAO, PAO1	0.416	ns
I1HGT7	Threonine synthase	thrC	2.660	ns
I1GZ41	Aldehyde dehydrogenase (NAD+)	E1.2.1.3	2.936	ns

*BYS/BYC represent the fold change of protein abundance from the comparison between treatment and control in Bai5, VS/VC represent the fold change of protein abundance from the comparison between treatment and control in Vao-9, ns represent the abundance change of the protein after salt stress was not significant*.

### Validation of the Transcript of DEPs by Real-Time Quantitative PCR

**Figure 6:**
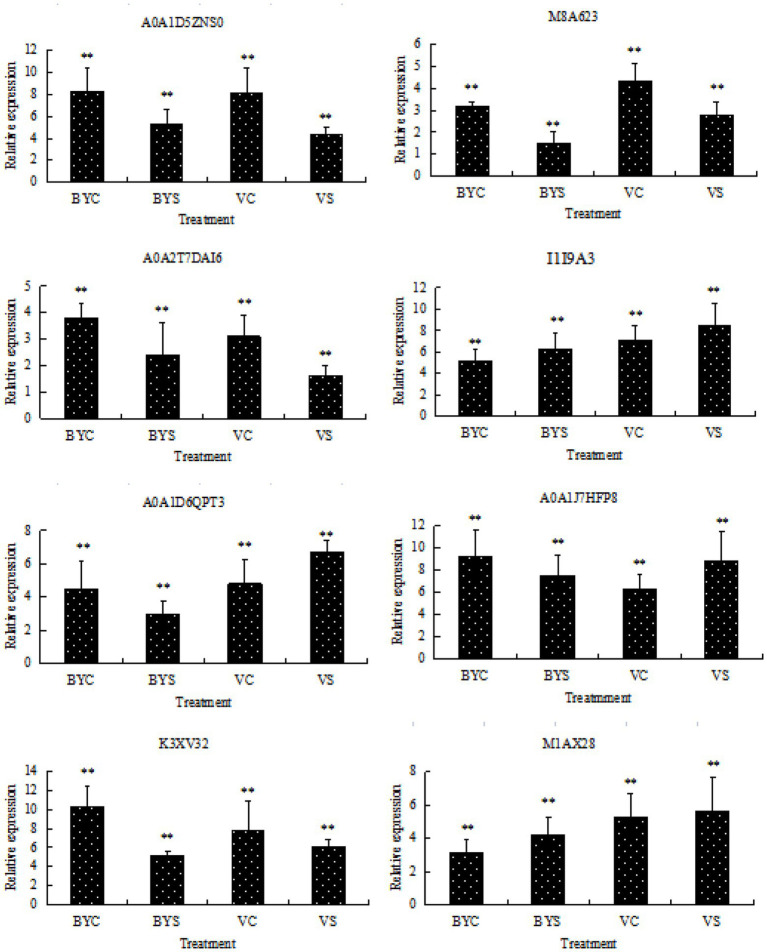
Gene expression analysis of DEPs by qRT-PCR. BYC and VC represent the untreated (CK) samples of Bai5 and Vao-9; BYS and VS represent the salt treated samples of Bai5 and Vao-9. Data given in form of mean ± SE, the significant difference determined by One-way ANOVA (Duncan’s test, ^**^p < 0.01).

Eight candidate differential proteins shared by the leaves of the two cultivars of oat were selected randomly and their transcription levels were determined by qRT-PCR as a reference for the verification of protein expression results. The designed primers are shown in Table 1, and the results are shown in Figure 6. Candidate protein abundance changes were consistent with transcript expression trends. This analysis can improve the confidence of the proteomic data.

## Discussion

### DEPs Involved in Carbohydrate and Energy Metabolism

Plants will quickly adjust their carbohydrate and energy metabolism to provide energy for resisting salt stress (Ghosh and Xu, 2014). The results obtained in this study indicated that the DEPs in the salt-tolerant variety Vao-9 are associated with carbohydrate and energy metabolism when compared with Bai5. This indicates that the carbohydrate and energy metabolism of Vao-9 undergoes very active and complex changes in the process of salt stress. These DEPs involve two metabolic pathways: pentose and glucuronate interconversions, and carbon fixation pathways in prokaryotes. The two pathways were up-regulated indicating that salt stress promotes the normal carbohydrate and energy metabolism of Vao-9 during the early salt stress response process of oats. UDP-glucuronic acid is a kind of nucleotide sugar, which is the precursor of the cell wall. Previous studies have reported that UDP-glucuronic acid is formed by UDP-glucose 6-dehydrogenase (UGDH), which catalyzes the production of UDP-glucose from UDP-glucose acid (Klinghammer and Tenhaken, 2007; Perner et al., 2016). AKR1 plays a vital role in various plant metabolic reactions including detoxification of aldehydes, secondary metabolism, osmotic biosynthesis, and membrane transport (Zhang and Shi, 2018). The up-regulated expression of these proteins is probably because Vao-9 enhances the glyco-conversion pathway to resist salt stress.

Aconitase A (ACO) is a key intermediate between catabolism and biosynthesis, and the changes in carbon flow at these branch points will affect crop yields and product formation. Previous studies have found that the over expression of PPC reduces the rate of glucose consumption and organic acid excretion (Chao and Liao, 1993). Vao-9 may regulate the metabolic flow between phosphate acetone acid and ACO through the over expressed PPC and phosphoenolpyruvate-protein kinase (ppdK), thereby becoming a critical energy generation pathway under salt stress.

### DEPs Involved in P+rotein Synthesis

Most of the DEPs identified in this study are associated with protein synthesis pathways. Ribosome is the main site of protein synthesis, and different kinds of ribosomal proteins play a vital role in translation, ribosomal structure, and biogenesis (Gong et al., 2017). Previous studies have reported that the overexpression of ribosomal protein results from this species maintaining a balance between protein synthesis and degradation by accelerating protein biosynthesis in response to salt stress (Frukh et al., 2020). Inconsistent with these results, the DEPs associated with ribosomes were not enriched in Bai5 and the ribosomal proteins in Vao-9 were all down-regulated, indicating that the salt tolerance mechanisms of plants are diverse. We speculate that the ribosomes may be programmed to be degraded in Vao-9 in order to reduce the cytoplasmic Na^+^ toxicity under salt stress, but not in Bai5. The results obtained after the analysis of physiological indicators indicated that the content of Pro in Bai5 increased with the increase of salt concentration, while it first decreased and then increased in Vao-9. Therefore, the accumulation in Bai5 is likely due to the conversion of other amino acids, while the accumulation in Vao-9 is probably the result of protein degradation. Heat shock protein (HSP) is considered to have the function of molecular chaperones. Several studies have reported that they are ubiquitous in animals and plants, and play an important role in the stress resistance of plants (Mayer and Bukau, 1998; Ahuja et al., 2010). Under various stresses, HSP can protect its target protein from denaturation, misfolding, and aggregation (Vierling et al., 1989; Santhanagopalan et al., 2018). Previous studies have found that the expression of HSPs in plants is affected by salt stress (Hamilton and Heckathorn, 2001), and HSPs in some plants can enhance stress tolerance when overexpressed in transgenic plants (Huang et al., 2019). However, the function and mechanism of HSP under adversity conditions has not yet been elucidated. Therefore, the expression pattern of oat HSPs detected and identified in this study can be used to explore the function of HSPs under adversity conditions.

The relationship between Ca^2+^ storage and signaling systems has been fully studied in Arabidopsis. Ca^2+^ is stored in several organelles including endoplasmic reticulum (ER), vacuoles, mitochondria, chloroplasts, and cell walls. Previous studies have shown that the ER plays an important role in regulating Ca^2+^ homeostasis despite the vacuoles being the main Ca^2+^ chelating sites in plant cells (Stael et al., 2012; Costa et al., 2018). The ER contains a variety of Ca^2+^ binding proteins such as molecular chaperone BiP, calnexin, and calreticulin (CRT). Among them, CRT is mainly responsible for the storage of Ca^2+^ in plants (Hassan et al., 1995; Mariani et al., 2003; Jia et al., 2009). A previous study reported that the over-expression of complete TaCRT1 cDNA or fragments of the domain that encodes the domain enhances tobacco’s tolerance to salt stress (Xiang et al., 2015). In this study, the Ca^2+^ content in Bai5 was more reduced after salt stress than in the salt-resistant variety (Vao-9). However, CRT had a higher expression, indicating that Bai5 needed to store Ca^2+^ through expressed CRTs to resist the harm caused by salt stress.

### DEPs Involved in Stress Defense and Other Stress-Responsive Proteins

It is known that glutamine synthetase (glnA) catalyzes the ATP-dependent condensation of glutamate and ammonia to produce glutamine (Liaw et al., 1995). Moreover, proline is a penetrant that overcomes pressure conditions, and glnA is involved in the synthesis of its biological precursors. A previous study reported that the overexpression of glnA in plants confers resistance to biotic and abiotic stresses (Hoshida et al., 2000), which is consistent with the results obtained in this study. Studies have shown that different plants can accumulate non-protein amino acids GABA under different stress conditions (including salinity; Kinnersley and Turano, 2000). GABA metabolism requires GAD enzyme, with pyridoal phosphate as a cofactor, to catalyze the decarboxylation of glutamate to GABA. The results obtained in this study indicated that glutamate decarboxylase (GAD) was up-regulated in Vao-9. This result when combined with existing studies in Arabidopsis indicate that the expression of GAD2 gene was enhanced within 24 h after NaCl treatment (Renault et al., 2010), which may be the performance of resistance to salt stress.

In addition, Glu decarboxylation is not the only way to synthesize GABA in plants because GABA can also be obtained through polyamine (PA) degradation. This process is carried out by amine oxidase (AOs), which is divided into polyamine oxidase (PAO) and two amine oxidase (DAO; Flores and Filner, 1985). A previous study has also proposed that the pathway generated by Pro involves non-enzymatic and enzymatic reactions to synthesize GABA under oxidative stress conditions (Signorelli et al., 2015). The results obtained in this study found that different DEPs exhibit tissue specificity in different resistant oat varieties, indicating that Bai5 and Vao-9 synthesize GABA through the above two different metabolic mechanisms, thereby resisting salt stress on oats. However, further studies should be conducted to determine the specific metabolic process.

## Conclusion

This is the first systematic report on the salt reaction mechanism in oat leaves with different salt tolerance based on proteomics analysis. The Label-Free method identified 2,631 salt-reactive proteins. Among these proteins, 262 DEPs changed significantly after 150 mmol L^−1^ salt treatment, and the changed proteins were mainly divided into three categories. From the results, we obtained tissue-specific information on the expression profiles of oat leaves with different salt tolerance. In the early salt stress response process, the salt-tolerant variety Vao-9 mainly enhances its carbohydrate and energy metabolism through the pentose and glucuronate interconversions, and carbon fixation pathways in prokaryotes, thereby reducing the damage caused by salt stress. In addition, the down-regulation of ribosomes expression and the up-regulated expression of HSPs and CRT were all achieved through the regulation of protein synthesis in response to salt stress, which did not change significantly in Bai5. However, GABA metabolism presents a different synthesis pattern in Bai5 and Vao-9. Therefore, the expression profiles of different salt-tolerant oats show that there is an interconnected but unique salt reaction mechanism in oats. Our comparative analysis of physiology and proteomics of different oat genotypes under salt stress will help in understanding the response process of different oat genotypes to salt stress. Therefore, the results obtained in this study will provide an important basis for further research on the underlying mechanisms of salt response and tolerance in oats and other plant species.

## Data Availability Statement

The original contributions presented in the study are publicly available. This data can be found here: iProx, IPX0004215000.

## Author Contributions

JL conceived and supervised the experiments. XC performed the experiments, contributed to data analysis, and wrote the paper. ZX, BZ, YY, JM, and ZZ gave valuable advice for the modifications of the paper. All authors contributed to the article and approved the submitted version.

## Funding

This work was supported by the National Natural Science Foundation of China (31560357) and the National Modern Agricultural Industry Technology System (CARS-08-B-5). Our sample testing data analysis was assisted by Beijing Novogene Technology Co., Ltd. (Beijing, China).

## Conflict of Interest

The authors declare that the research was conducted in the absence of any commercial or financial relationships that could be construed as a potential conflict of interest.

## Publisher’s Note

All claims expressed in this article are solely those of the authors and do not necessarily represent those of their affiliated organizations, or those of the publisher, the editors and the reviewers. Any product that may be evaluated in this article, or claim that may be made by its manufacturer, is not guaranteed or endorsed by the publisher.
